# Continuous Glucose Monitors Among Adults With Type 2 Diabetes Mellitus in the Primary Care Setting: Qualitative Study Informed by Technology Acceptance Model and Health Belief Model

**DOI:** 10.2196/73446

**Published:** 2025-12-30

**Authors:** Michelle I Knopp, Ann Marie Castleman, Anna Schwarz, Jamarin Belger-Wallace, Mercedes Falciglia, Aleona Zuzek, Eneida Mendonca

**Affiliations:** 1 Department of Pediatrics, College of Medicine, University of Cincinnati, 3333 Burnet Ave, MLC 7024, Cincinnati, OH, 45229, United States, 1 513-803-4720; 2 Department of Biostatistics, Health Informatics and Data Science, College of Medicine, University of Cincinnati, Cincinnati, OH, United States; 3 Division of Biomedical Informatics, Cincinnati Children's Hospital Medical Center, 3333 Burnet Ave, MLC 7024, Cincinnati, OH, United States; 4 Division of Hospital Medicine, Cincinnati Children's Hospital Medical Center, Cincinnati, OH, United States; 5 Department of Internal Medicine, College of Medicine, University of Cincinnati, 231 Albert Sabin Way, Cincinnati, OH, United States; 6 Evaluation Services Center, College of Education, Criminal Justice, Human Services and Information Technology, University of Cincinnati, Cincinnati, OH, United States; 7 Department of Enviornmental and Public Health Science, College of Medicine, University of Cincinnati, Cincinnati, OH, United States; 8 Endocrinology, Advocate Health, Charlotte, NC, United States

**Keywords:** continuous glucose monitors, CGM, primary care, qualitative research, Technology Acceptance Model, type 2 diabetes mellitus

## Abstract

**Background:**

Continuous glucose monitors (CGM) reduce the burden of glycemic monitoring and improve glycemic control, quality of life, and decreased health care use. Despite expanded insurance coverage and adoption, barriers remain, especially in primary care. Existing research largely evaluates specific populations or interventions, leaving limited insight into the broader primary care experience.

**Objective:**

This study aims to examine the experiences of adults with type 2 diabetes mellitus (T2DM) using CGM in primary care, guided by the Health Belief Model and Technology Acceptance Model.

**Methods:**

This qualitative study included in-person semistructured sessions (interviews or a focus group), surveys, and electronic health record data. Participants were recruited from 3 urban primary care (internal medicine and internal medicine-pediatrics) clinics affiliated with a large academic health system in Southwest Ohio, United States, with high rates of public insurance (Medicare or Medicaid). Eligible participants were adults (≥18 y) with T2DM and a CGM prescription. Data were analyzed using theme generation guided by directed content analysis in MAXQDA (VERBI Software GmbH) with codes derived from Health Belief Model and Technology Acceptance Model constructs. Survey data were used to triangulate to enhance validity.

**Results:**

Overall, 16 participants (interviews: n=12; 1 focus group: n=4) were recruited for the study with a mean age of 56.9 (SD 10.5) years. In total, 69% (11/16) identified as Black, 100% (16/16) as Non-Hispanic, and 69% (11/16) as female, and 94% (15/16) used public insurance. Six themes emerged: disease susceptibility, disease severity, influential drivers, perceived ease of use, perceived usefulness, and attitude toward using CGM. All participants found CGM helpful and would recommend it to others. While affirming numerous barriers well-described in other populations, this study uniquely describes the burden of comorbidities, the trust in CGM data compared to glucometer-based monitoring, and the reliance on receivers to use CGM technology in this patient population.

**Conclusions:**

CGM is valued by adults with T2DM in primary care, yet barriers remain. Tailored support for initiation, troubleshooting, and education (especially alarm management and data interpretation) is needed. These insights can inform scalable strategies to enhance CGM use and experience in primary care.

## Introduction

### Background

Continuous glucose monitors (CGM), approved by the US Food and Drug Administration in 1999 [1], use a subcutaneous sensor to measure interstitial fluid glucose, reducing monitoring burden and revealing fluctuations that might otherwise be missed. This technology reduced the need for painful fingerstick testing with glucometers that require several steps and supplies [2]. CGM use improves glycemic control [3-10], reduces hypoglycemia and variability, enhances the quality of life [5], and lowers health care use and costs [6-10]. Since diabetes affects 10.5% of the global adult population [11], remains the seventh leading cause of death in the United States [12], and is associated with substantial disease-related distress [13], harnessing the potential of CGM and other emerging technologies may be key to improving outcomes.

Previously, broad CGM use was limited in patients with type 2 diabetes mellitus (T2DM) by a variety of factors. Access to specialized endocrinology care [14] is limited, and despite increased prescribing [15], primary care staff and providers need additional training to be comfortable with the technology [16]. Additionally, CGM use was previously emphasized for patients with insulin-dependent T2DM, and insurance coverage was limited. Policy changes have expanded CGM coverage and accessibility [17,18], making broader CGM use, especially in primary care, more realistic. Many patients with T2DM and prediabetes are managed in primary care settings, and CGM can serve as a patient education tool [19].

In order to maximize CGM use for T2DM in primary care, more work is needed. To date, little is known about the adult patient experience using CGM as part of primary care T2DM management. Without this knowledge, barriers to optimal use cannot be adequately addressed. Recent qualitative work in the United States on outpatient CGM use focused on youth-onset T2DM [20-22], Hispanic adults [23-25], federally qualified health centers [25], or CGM use in combination with other interventions [26]. More work is needed to better understand the common barriers to CGM use in primary care and the issues that primary care providers and staff must help patients overcome. For instance, CGM cost, wearability, discomfort, or fear related to sensor insertion, lack of support, need for guidance on data interpretation, educational needs, and other physical or social factors may all influence CGM use [27,28].

We sought to identify disease- and technology-specific factors influencing CGM use in primary care. Therefore, to guide this work, we chose the Health Belief Model (HBM) [29,30] and the Technology Acceptance Model (TAM) [31]. HBM explains health behaviors through constructs, such as perceived susceptibility, perceived severity, perceived benefits, perceived barriers, self-efficacy, and cues to action. The TAM complements HBM by addressing technology adoption through external variables, perceived usefulness and ease of use, attitude toward using, behavioral intention to use, and actual system use. Combining TAM and HBM enables a holistic evaluation of factors influencing CGM use.

### Objective

This study aims to understand the perspective of adults with T2DM using CGM in primary care.

## Methods

### Study Design

This is a qualitative study using directed content analysis examining the experiences of adults with T2DM using CGM in primary care in the United States, using in-person sessions (interview or focus group) with a survey; diabetes-related health data were extracted from the electronic health record (EHR) for each respective participant. We sought to complete 3 focus groups in addition to individual interviews. Study design was guided by the TAM [31] and HBM [30] with an interpretivism paradigm [32]. The interview guide in Multimedia Appendix 1 was internally developed and included questions adjusted to meet fifth-grade reading level. It was internally reviewed but not pilot tested. The interview guide was submitted and approved by the University of Cincinnati Institutional Review Board.

The COREQ (Consolidated Criteria for Reporting Qualitative Research) checklist [33] is available in Checklist 1.

### Setting

We recruited participants from 3 urban primary care clinics affiliated with a large academic health care system in Southwest Ohio, United States: (1) an internal medicine resident clinic, approximately 7000 patients, 30% have diabetes (80% have hemoglobin A_1c_ [HbA_1c_] <8%, ~18% use CGM); (2) an internal medicine attending clinic, approximately 3500 patients, 24 % have diabetes (81% have HbA_1c_ <8%); and (3) a combined attending and resident internal medicine-pediatrics clinic, approximately 6000 patients, 11% have diabetes (84% have HbA_1c_<8%). These clinic populations are approximately 70% publicly insured (Medicaid, Managed Medicaid, Medicare, or Managed Medicare) and 25% privately insured. The participating clinics were purposively selected due to high public insurance rates, a known marker in the United States for lower socioeconomic status. All clinics are supported by an in-house pharmacy team and nurse practitioners. Providers in all 3 clinics routinely care for patients with T2DM. All sessions took place in-person in an on-site location used for diabetes education.

### Recruitment

Participants were recruited by MIK via convenience sampling from the 3 purposively selected clinics. We recruited patients using flyers posted in clinics, announcements to primary care providers and pharmacists, and identifying eligible participants on clinic schedules. MIK contacted potential participants via patient portal messages and phone calls. Eligibility criteria included a diagnosis of T2DM, CGM prescription, 18 years and older, nonpregnant, and the ability to participate in a 1-hour in-person session in English. Participants were assigned to a session type based on their availability.

### Research Team

The research team included an internal medicine primary care physician with clinical informatics training (MIK), a psychology doctorate student (AMC), an internal medicine resident (AS), an undergraduate student (JBW), a senior endocrinology attending (MF), an endocrinology fellow (AZ), and a senior physician scientist in biomedical informatics (EAM). Some team members have first-hand experience monitoring glucose via CGM and glucometers. This work is informed by the belief that chronic disease leads to a significant burden for patients and that technology can help reduce that burden. However, often technical, societal, structural, and individual factors prevent technology from optimizing disease management. This study was developed from MIK’s experience working with patients in primary care and recognition that, while access to CGM was improving, the clinical impact described in the literature seemed to be lagging. MIK served as a supervising physician in the Internal Medicine clinic, where most patients were recruited but did not provide direct care to the patients recruited for this study. The team was supported by the University of Cincinnati Evaluation Services Center (ESC), which provides knowledge, expertise, and experience to serve as an evaluation and applied research partner. ESC provided interviewer training to MIK and supported the development of the protocol, codebook, and overall evaluation. Initial interviews included virtual support from the ESC to ensure quality and provide training for the primary interviewer (MIK).

### Data Collection

We conducted 1-hour in-person semistructured sessions (interviews and a focus group). Interviews were conducted by MIK, while the focus group was facilitated by the ESC (EM and AMC). No repeat interviews were conducted. Sessions were audio-recorded and transcribed via conferencing software (Microsoft Teams or Zoom). Transcripts were manually reviewed and edited by study staff. Identifying information was removed. As most sessions included only 1 study interviewer, field notes were limited. No nonparticipants were present during sessions. Transcripts were not returned to patients. Recruitment was terminated when data saturation [34] was achieved, as determined by no new themes, insights, or perspectives emerging from subsequent interviews (MIK and EAM).

Participants completed a paper survey after the session that included questions around perception of CGM using a Likert scale [22], usage habits, and social determinants of health, which was later transcribed into REDCap (Research Electronic Data Capture; Vanderbilt University) by MIK. EHR data were manually extracted via chart review to a REDCap form by MIK. Fields included demographics (age, ethnicity, race, sex, insurance type, and smoking), medical history (length of diabetes diagnosis, complications, and comorbidities), medications, appointment information (number of primary care physician, emergency department, and endocrinology visits in the last year), laboratory data (estimated glomerular filtration rate, cholesterol, HbA_1c_ at first CGM prescription, and most recent HbA_1c_), and completion status of health care maintenance tasks (eye exam, HbA_1c_, urine protein, and foot exam). Study data were managed using REDCap [35,36].

### Statistical Analysis

Transcripts were coded using MAXQDA [37] (VERBI Software GmbH) and analyzed using a qualitative content analysis [38,39]. A priori codes were based on TAM and HBM, with inductive codes added during review of the first 3 transcripts (MIK and AMC; Multimedia Appendix 2). A total of 2 team members independently coded each transcript. Coding discrepancies were resolved by discussion and consensus. Theme generation was guided by directed content analysis [40] starting with the a priori codes. Inductive codes were evaluated (JBW) and combined with larger themes as appropriate. Data were examined for patterns within and across codes to identify potential themes and subthemes. Iterations of themes and subthemes were validated by team review (MIK, ACZ, AS, JBW, and EAM). Member checking was not performed. An audit trail documented coding and theme development. To strengthen rigor, we used methodological triangulation by comparing survey data on CGM perceptions with quantitative themes. Survey and EHR data were aggregated by session type. Categorical data are reported as counts, percentages; continuous data as mean, standard deviation, range (minimum - maximum).

### Ethical Considerations

This study was approved by the University of Cincinnati Institutional Review Board (study number 2023‐0593). The protocol was deemed to be no greater than minimal risk. Written informed consent was obtained from all participants, including disclosure of the study goals. Participants could opt out at any time. Non-essential identifying information has been removed for publication. Participants were informed that direct quotes from the session may be used. Techniques to reduce power imbalances and promote equitable participation were used [38,41], such as using first names, removing badges, and wearing casual business clothing. Participants were compensated with a US $50 cash card.

## Results

### Overview

A total of 46 candidates initiated or received correspondence from the principal investigator; 16 participants (12 interviews and one 4-person focus group) were enrolled between December 2023 and June 2024 (Table 1)**,** when data saturation was reached. Reasons for nonparticipation included inability to be reached, scheduling conflicts, and missed appointments.

**Table 1.** Participant characteristics.

Characteristics	Interview (n=12)	Focus group (n=4)	Overall (N=16)
Age (years), mean (SD), range	57.3 (8.6), 44-69	56 (16.8), 40-75	56.9 (10.5), 40-75
Race, n (%)			
White or Caucasian	4 (33)	0 (0)	4 (25)
Black or African American	8 (67)	3 (75)	11 (69)
Multiracial	0 (0)	1 (25)	1 (6)
Ethnicity (interview and focus group total), n (%)			
Non-Hispanic	12 (100)	4 (100)	16 (100)
Hispanic	0 (0)	0 (0)	0 (0)
Sex, n (%)			
Female	8 (67)	3 (75)	11 (69)
Male	4 (33)	1 (25)	5 (31)
Insurance, n (%)			
Commercial	1 (8)	0 (0)	1 (6)
Medicare	4 (33)	2 (50)	6 (38)
Medicaid	7 (58)	2 (50)	9 (56)
Have device, n (%)^a^			
Desktop or laptop	5 (42)	2 (50)	7 (44)
Smart phone	10 (83)	2 (50)	12 (75)
Tablet	6 (50)	0 (0)	6 (38)
None	1 (8.3)	0 (0)	1 (6)
Internet access at home, n (%)^a^			
Cellular plan	5 (42)	2 (50)	7 (44)
Broadband	10 (83)	2 (50)	12 (75)
Primary mode of transportation, n (%)			
Personally owned car	7 (58)	3 (75)	10 (63)
Insurance provided transportation service	2 (17)	0 (0)	2 (13)
Public transportation	3 (25)	1 (25)	4 (25)
Diabetes medications, n (%)^a^			
Basal insulin	6 (50)	4 (100)	10 (62)
Bolus insulin	4 (33)	4 (100)	8 (50)
Metformin	5 (42)	2 (50)	7 (44)
SGLT2^b^	6 (50)	1 (25)	7 (44)
GLP1^c^	8 (66)	1 (25)	9 (56)
Thiazolidinedione	1 (8)	0 (0)	1 (6)
Statin	11 (92)	3 (75)	14 (88)
Complications, n (%)^a^			
Neuropathy	4 (33)	2 (50)	6 (38)
Nephropathy	2 (17)	1 (25)	3 (19)
Cardiovascular	5 (42)	0 (0)	5 (31)
Retinopathy	1 (17)	2 (50)	3 (19)
Years of CGM^d^ use, mean (SD), range	1.4 (0.8), 0.1-3	3.4 (1.9), 1.5-5.2	1.9 (1.4), 0.1-5.2
Sensor type, n (%)^a^			
FreeStyle Libre 14 Day	1 (8)	0 (0)	1 (6)
FreeStyle Libre 2	5 (42)	2 (50)	7 (44)
FreeStyle Libre 3	2 (17)	1 (25)	3 (19)
Dexcom G6	3 (25)	1 (25)	4 (25)
Dexcom G7	4 (33)	0 (0)	4 (25)
Connected device, n (%)			
Receiver	9 (75)	3 (75)	12 (75)
Phone	3 (25)	1 (25)	4 (25)
Years since DM^e^ diagnosis, mean (SD), range	12.2 (6.1), 3-22	12.8 (2.2), 10-15	12.3 (5.4), 3-22
HbA_1c_^f^ at first CGM prescription, mean (SD), range	9.2 (3.3), 5.2-16.9	9.3 (2.8), 6.3-13	9.2 (3.1), 5.2-16.9
Most recent HbA_1c_, mean (SD), range	7.7 (1.7), 5.4-10.4	8.6 (1.2), 7.6-9.7	7.9 (1.6), 5.4-10.4
Endocrinology appointment in last year?, n (%)	2 (17)	0 (0)	2 (13)

a Select all that apply, therefore columns may add up to more than 100%.

b SGLT2: sodium-glucose cotransporter 2.

c GLP1: glucagon-like peptide-1.

d CGM: continuous glucose monitoring.

e DM: diabetes mellitus.

f HbA_1c_: hemoglobin A_1c_.

The mean age was 56.9 (SD 10.5; range 40‐75) years, 69% (11/16) identified as Black, 100% (16/16) identified as non-Hispanic, and 69% (11/16) as female. Most (15/16, 94%) use public insurance. The mean diabetes duration was 12.3 (SD 5.4; range 3‐22) years, with initial HbA_1c_ of 9.2% (SD 3.1; range 5.2‐16.9) and most recent HbA_1c_ of 7.9% (SD 1.9; range 5.4‐10.4). Only 2 participants had an endocrinology visit in the past year. Ten participants used insulin. Mean CGM use was 1.9 (SD 1.4; range 0.1‐5.2) years. Devices included FreeStyle Libre 14 Day, FreeStyle Libre 2, FreeStyle Libre 3, Dexcom G6, and Dexcom G7. Most (12/16, 75%) used a receiver rather than a phone for glucose viewing.

Survey results showed all participants strongly agreed that CGM was useful and would recommend it to a friend. Additionally, 93% (15/16) strongly agreed that CGM was helpful, and 87% (14/16) reported (agreed or strongly agreed) a positive experience using CGM.

### Qualitative Results

Informed by TAM and HBM, 6 themes emerged (Table 2): disease susceptibility, disease severity, influential drivers, perceived ease of use, perceived usefulness, and attitude toward using CGM. Triangulation with survey data confirmed that despite challenges, participants valued CGM and would recommend it.

**Table 2.** Summary of themes and subthemes.

Themes	Subthemes
Disease susceptibility	Diagnosis Emotional response to diagnosis Contribution of lifestyle
Disease severity	Unstable disease control Medication burden Fear of complications
Influential drivers	Insurance coverage Support system Motivation Information sources Comorbidities
Perceived ease of use	Model differences Initiation Sensor Receiver Alarms Skin reaction
Perceived usefulness	Access to continuous data Trust in the data Data sharing
Attitude toward using CGM^a^	Empowering disease management Recommend to others Improvement over Glucometer-based monitoring Further opportunities

a CGM: continuous glucose monitor.

### Disease Susceptibility

Disease susceptibility, a component of the HBM, refers to a person’s perception of the risk of acquiring a disease. Subthemes include context of diagnosis, emotional response, and contribution of lifestyle factors (Table 3).

**Table 3.** Disease susceptibility and severity subthemes and pertinent quotes.

Themes and subthemes	Quotes
Disease susceptibility	
Context of diagnosis	“It's probably been 15 years, since more of the like the pre diabetes kind of ramping up into the Type 2. So they're slowly watching of the A1C kind of going up.” [Participant 9]
Emotional response to diagnosis	“I've been pre diabetic for a very long time and I guess I kind of jinxed myself by telling myself I'm will eventually be a diabetic just because everybody in my family has type 2 diabetes.” [Participant 7] “It was a shock when I found out I had diabetes, you know, cause I think I'm the first person in our family.” [Participant 1]
Contribution of lifestyle	“I'm just assuming that the key is in not being as active as I used to be led to the sugar diabetes.” [Participant 8] “I was borderline diabetic for a very long time before they actually made the full diagnosis… because I was overweight child, overweight teenager.” [Participant 12]
Disease severity	
Unstable disease control	“I have moments where I just throw it up in the air and say screw it. You know especially if I’m real depressed or upset about something, I just don’t care. I’ll be honest, just lose it for a while.” [Participant 2]
Medication burden	“I haven’t been the best at the medicine... I just don’t want to take [them]. I’ll wrestle with myself for a while… I hear all these side effects that like that it’s more problem taking it then if I don’t.” [Participant 5] “I wish you guys could be a guinea pig for one day to see what patients go through and that’s what gets them discouraged is the side effects. Yep, I think that discourages a lot of people because people don’t have the patience.” [Participant 1]
Fear of complications	“I have a neuropathy in my feet. When I take my shoes off, my feet tingles. It feels like somebody stabbing a 1000 needles. Horrible” [Participant 6]

Participants described varied contexts of diagnosis, including specific events, family history, and progression from prediabetes. Reactions to diagnosis ranged from denial and surprise to inevitability, especially among those with a family history. Additionally, participants recognized the impact of lifestyle factors such as obesity and limited exercise on diabetes development.

### Disease Severity

Disease severity, a component of HBM, captures a person’s feelings on the seriousness and consequences of diabetes. Subthemes include the unstable disease control, medication burden, and fear of complications (Table 3).

Participants noted the unstable nature of disease control, with difficulties such as “sugar addiction” and the potential for life events to derail progress. Despite the focus not being on medications, participants discussed side effects, drug shortages, dose uncertainty, and distrust leading to self-discontinuation. Fear of complications, such as blindness, neuropathy, and death due to poor diabetes control, was prevalent.

### Influential Drivers

Influential drivers, combining cues to action from HBM and external variables from TAM, look at drivers that improve diabetes management and the acceptance of CGM. Subthemes include insurance coverage, support systems, motivation, information sources, and comorbidities (Table 4).

**Table 4.** Influential drivers subthemes and quotes.

Influential drivers	Quotes
Insurance coverage	“The only thing I don't like … it's not the [CGM]^a^, it is the insurance company. The insurance company that I have, they won't pay for [certain brand of CGM]. The one I have, it's not compatible to my phone and it won't alert me when it's going low.” [Participant 3]
Support system	“I love [primary care doctor] she’s excellent. She gets on my case, which I need it cause sometimes and I know better, I’m a nurse. I know what [diabetes] does to you, but sometimes you know I drop the ball a little bit and then maybe after just short time, I get back on the ball again.” [Participant 2] “Well, I like her [Pharmacist] a lot better than the doctors trying to explain it because this is not their forte and she seems to know what she’s talking about… She’s patient and she explains things to you know, and she knows me.” [Participant 1]
Information sources	“I know there’s commercials on TV, but I don’t know if they [people with diabetes] really understand what the commercials is telling them. It would be a big improvement. You don’t have to stick your fingers no more. How it lets you know when your blood sugars are high or low. I don’t think everybody, all diabetics, really understand that.” [Focus Group Participant 2] “Sometimes I look on YouTube. I don’t know how accurate it is, but I look at it and I say that might work…I have just been trying to find the things that work for me.” [Participant 5]
Comorbidities	“And it’s hard for me to see. Yes, with that thing [receiver] is like the numbers kind of a little bit bigger than that [on phone].” [Participant 4]

a CGM: continuous glucose monitor.

Participants highlighted the critical role of insurance coverage, noting they couldn’t use CGM without it and that device choice was often dictated by coverage. Support systems, including family, community, and health care team members, were also significant. Motivation for disease control stemmed from the desire to live for loved ones, manage symptoms, and optimize HbA_1c_. Information about CGM came from advertisements, health care teams, family and friends, and YouTube (Google; 3/16, 19%). Participants reported comorbidities in mental health, cardiovascular, musculoskeletal, neurological, vision, and obesity, with mental health and vision preventing optimal CGM use.

### Perceived Ease of Use

Perceived ease of use, from the TAM, examines how easy or difficult individuals find CGM to use. Subthemes include model differences, initiation, sensor, receiver, alarms, and skin reactions (Table 5).

**Table 5.** Perceived ease of use subthemes and quotes.

Perceived ease of use	Quotes
Initiation	“I've had nobody to really teach me anything about it. Everybody's too busy.” [Participant 2] “The first time I didn't understand it. When I first did it. So when it fell off, I said I don't know what to do. And my son ain't here to stick me. So I just let it go.” [Participant 8] “My phone has told me “your phone is too old to download apps.” How embarrassing? But I can't afford to get one right now, so I'm just sticking with it [receiver].” [Participant 1]
Sensor	“I’ve had a couple fall off like taking off a shirt” (Participant 2) “I’m good with the arm because I stick myself in the stomach with my insulin.” [Participant 3]
Receiver	“I had a few issues with it cause the machine wants to act up or keep saying like I can’t locate you”. [Participant 1] “Well, first it was a problem because now I got to carry this. I got to carry this thing, you know, I’m forgetting it when I come out the door. Now I can’t check it if I don’t have the monitor. Tried hooking it up to the phone initially. That didn’t work.” [Participant 5] “And it’s hard for me to see. Yes, with that thing is like the number’s kind of a little bit bigger [on receiver than on phone] than that, trying to …connect it and programming all that. That’s kind of frustrating to me.” [Participant 4]
Alarms	“I stopped using it cause of the dinging so much, I cut my phone off and don’t wanna hear.” [Participant 8]
Skin reaction	“I noticed that I was using the same area, probably a little bit more than I should have. I only say that because you know the entry point starts to show up. It doesn’t get a chance to heal before you’re putting another one either right over top of it or right in the same area where it’s at. So having to stop using it temporarily to give that area a chance to heal up and use another area, or go back to the sticking for a short period of time.” [Participant 5]

Participants who used multiple models (19%, 3/16) reported significant differences and strong preferences. Initiation challenges included logistics, troubleshooting, fear of needles, and phone compatibility issues. Sensor barriers involved a lack of application help, optimal location, and water resistance. Receiver issues included connectivity problems, frequent misplacement, and a preference for receivers over phones. Alarms caused significant annoyance, leading some to discontinue use. Various skin reactions required troubleshooting.

### Perceived Usefulness

Perceived usefulness, from TAM, describes how individuals believe CGM helps manage diabetes. Subthemes include access to continuous data, trust in the data, and data sharing (Table 6).

**Table 6.** Perceived usefulness subthemes and pertinent quotes.

Perceived usefulness	Quotes
Access to continuous data	“This meter, it helps me keep track of myself and where I am. Honestly, I have to be honest with myself where I am at all times. 24 hours a day. It keeps me on point. Period. It's just plain and simple.” [Participant 6] “It helped me a lot because it made me learn a lot from the [CGM]^a^… I had it for so long that I can tell when my sugar is high or low.” [Focus Group Participant 1] “This [CGM] is better than, almost better than … my heart defibrillator because it's …keeping me alive, but this [CGM] has helped keeping me from going into a coma or getting that to where I have to come to the hospital. I can pretty much take care of it [diabetes]…. this little device here I love it. I just love it.” [Participant 3]
Trust in the data	“it’s more accurate to me because when I used to stick my fingers, it would say one thing and then I’ll check it on another finger and it says something totally different. So if I check it two or three times within the couple minutes, it’s saying the same thing and unless its changed. It’s not a big change but I really love it. I love it.” [Participant 3]
Data sharing	“I like the fact that the doctors can see it. I can’t reiterate that enough. It helps when they can read your blood sugars … then they can figure out what medications to prescribe.” [Participant 12] “My son, he got the notification on his phone too. So anything I ain’t doing right, he get notified. He either call me first or come on out“. [Participant 8]

a CGM: continuous glucose monitor.

Participants valued continuous data for constant feedback, learning, responding to extreme values, optimizing medications, and behavior change. They noted improved glucose values and better understanding of their body’s reactions, which kept them motivated. Trust in CGM data was higher compared to traditional glucometer-based monitoring. While few (13%, 2/16) mentioned CGM-specific metrics, most discussed HbA_1c_ and current glucose values. Data sharing with family and providers was also highly valued.

### Attitude Toward Using CGM

Attitude toward using CGM, derived from the TAM, captures an individual’s overall views on CGM for diabetes management. Subthemes include empowering disease management, recommendations to others, improvements over glucometer-based monitoring, and further opportunities (Table 7).

**Table 7.** Attitude toward using continuous glucose monitor subthemes and quotes.

Attitude toward using CGM	Quotes
Empowering disease management	“I can appreciate the [CGM]^a^. I really do. It's changed my life in so many ways. It’s helped me to live a better, smoother life, you know, so that I can stay on track with myself and I can be good for my kids, my wife and my family.” [Participant 6] “I stopped wearing the monitor for a while. That's when I fell off the bandwagon again, because you don't have that, that constant reminder of being able are you in the green, are you in the yellow, are you in the red? I definitely attribute compliance to that continuous monitor.” [Participant 9] “[if someone starts CMG] they're going to learn so much about the diet and how their body responds to food.” [Participant 9]
Recommend to others	“Don’t hesitate. Get it. Go with your first thought. Get it. Get it. Get it.” [Participant 3]
Improvement over Glucometer-based monitoring	“I’m a little happy because my fingers are not sore and I can do you can do what you love to do, which is I like to sew. I like to play games on my tablet and if my fingers are sore, I’m not gonna do all that... I don’t have to worry about sticking myself. I can just go grab my little meter and scan and there it is and not have to sit and figure out which finger I wanna use and all that so it’s made a little happier…I don’t have to worry about… do I have enough test strips or you know, is my meter working or do I need batteries or anything like that. With this all you do is charge it and that’s it.” [Participant 3]
Further opportunities	“14 days is good, but if it [sensor] could last about 30 days, I’ll be on board with that.” [Participant 3] “Well, I know that it’s not going to be able to do this, but help me with my self-discipline.” [Participant 7]

a CGM: continuous glucose monitor.

Participants felt empowered by CGM, as it allowed easier glucose tracking and understanding of food impact. They adjusted their diets by reducing sweetened beverages, candy, simple carbs, and certain fruits, while increasing fresh produce, grains, diet soda, water, fiber, and lean proteins. All participants highly recommended CGM to others, noting reduced stress in diabetes management. They preferred CGM over traditional glucometer-based monitoring due to no pain, fewer supplies, and less stress. Participants had limited suggestions for future CGM improvements, but desired longer-lasting sensors and more concrete advice around diet and self-motivation.

## Discussion

### Principal Findings

In this study, we gained insight into the experience of adults with T2DM using CGM in primary care, guided by the HBM and TAM. Participants found that CGM was helpful, empowered them to manage their disease, was an improvement over glucometer-based monitoring, and they would recommend it. Disease susceptibility and severity appeared to motivate diabetes management and influence attitudes toward monitoring blood glucose. Influential drivers include insurance coverage, support systems, motivation, information sources, and comorbidities. The perceived ease of using the technology is related to CGM model differences, initiation process, sensors, receivers, alarms, and skin reactions. Perceived usefulness centers on continuous data, trust in readings, and data sharing. Attitude toward using CGM was generally positive. Our results confirm barriers noted in other populations and uniquely demonstrate the role comorbidities play in interfering with optimal use, strong trust in CGM data versus glucometer-based monitoring, and heavy reliance on receivers rather than phones among adults with T2DM using CGM in primary care.

### Comparison With Previous Work

#### Disease Susceptibility and Severity

Those with a family history of diabetes expressed a sense of inevitability about their diagnosis, underscoring the need for early intervention and opportunities for CGM to alter disease trajectory [42]. Since disease severity influences monitoring approaches, understanding a patient’s disease experience is critical for guiding CGM discussions.

#### Influential Drivers

This work demonstrates multiple system- and individual-level factors affecting CGM use. Insurance coverage is a well-documented barrier [43-45], particularly for certain populations [27]. Adherence and cost also vary by CGM source (pharmacy vs durable medical equipment supplier) [46]. Participants emphasized that diabetes is only 1 component of their daily life, shaped by support systems and comorbidities. The importance of support systems noted in youth-onset T2DM [20] and in Hispanic adults [24] holds true for the general primary care population.

Limited in the literature is how comorbidities impact optimal CGM use. While participants in this study highlight numerous comorbidities, vision and mental health directly impact CGM use. Patients report difficulty reading values due to poor vision. Mental health and diabetes distress often prevent participants from fully engaging in CGM use. Recognizing and intervening in these factors is critical for supporting patients.

This work also highlights numerous sources that expose patients to health information (TV advertisements, health care team, family, friends, and YouTube), identifying potential avenues to share CGM-related information with patients beyond the clinic setting.

#### Perceived Ease of Use

Our study offers insight into usability challenges across CGM models and factors influencing adoption. Optimal use requires matching the best CGM model to each patient, supporting initiation, enabling troubleshooting, and leveraging alarm benefits. Because models differ in capabilities, informed decision-making is essential for acceptance. Some tools have been developed for people with type 1 diabetes [47]; however, due to the rapidly evolving nature of diabetes technology, maintaining tools to support informed decision-making remains difficult.

Notably, most participants (12/16, 75%) used receivers and preferred receivers. This significantly impacts the ability to use CGM for population health management asynchronously, as receivers neither automatically nor remotely upload data for clinician review, necessitating in-person encounters. This has not been previously described in the literature. In our clinical workflows, the CGM Order Set defaults to prescribing patients a receiver, which may have some impact on the overall high receiver use. However, participants described sensors not being compatible with the phone they have, both as a function of the age and model of their phone, and of insurance coverage for sensors. Many participants needed replacement receivers due to loss or damage, so pathways for replacement receivers are critical for consistent CGM use. Therefore, for successful CGM use in primary care, clinics will need to develop support for receivers, troubleshoot phone compatibility, and identify pathways to compatible phones for patients.

Despite the difficulties in initiation, current literature does not provide optimal strategies to address this barrier. Some programs use an interprofessional team to assist with CGM initiation [48], but these have not been widely implemented.

As with any technology, end users need ways to solve problems. This mirrors findings from CGM initiation in adults with type 1 diabetes who expressed a desire for support during initiation (insertion, adhesive, and common pitfalls), guidance on contacting the manufacturer’s customer support, data management strategies, attention to the mental and emotional side of CGM use, real-time troubleshooting advice, and access to advice from other users [49]. It is important to recognize the large treatment team involved in a patient’s care, including the primary care physician, endocrinologist, diabetes educator, pharmacist, insurance company, durable medical equipment supplier, and vendor. Navigating this team to get the right questions to the right person can be challenging, especially as only a few team members in primary care are often fully familiar with the technology and troubleshooting pathways.

Alarm burden [50] continues to hinder optimal use and leads to discontinuation for some participants. Research shows that high glucose alarms on CGM improve glycemic control [51]. Additionally, CGM education has been shown to enhance the use of low glucose alarms [52], suggesting the potential impact of CGM alarm education; however, best practices for primary care have not yet been developed.

#### Perceived Usefulness

The impact of any technology depends on the understanding of the user. Challenges in T2DM self-management include comprehension, skill development, and leveraging personal strengths [53]. Some participants reported difficulty interpreting numerical data, relying instead on alarms or color indicators. This highlights the need to enhance patient education and alarm management to optimize CGM use.

A recent community project demonstrated that CGM has the opportunity to make the invisible visible, support effective decision-making, enhance self-efficacy, diabetes-related diet modification, changes in physical activity, and changes in medication taking [54]. These findings were reinforced in this study with similarly identified subthemes.

Notably, unlike other work that reports privacy concerns [25] and lack of trust in the CGM data [24], participants in this study trusted CGM more than the variability of glucometer-based monitoring. Participants liked having only 1 value rather than trying to reconcile different values around the same time from glucometer-based monitoring due to blood from different fingers or the influence of contaminants on fingers. While CGM accuracy has improved, there remains a 15% variability around 100 mg/dL [55], which participants do not seem to fully appreciate. While patient trust in data is encouraging, ensuring that patients are not misguided will be critical to ensure safety.

This work is part of the broader effort to optimize the clinical use of CGM. The Integration of Continuous Glucose Monitoring Data into the Electronic Health Record project [56] developed standards for incorporating CGM data into clinical practice, which are now being used [57]. Opportunities to improve CGM effectiveness include education, modified EHR, team-based approaches, expanded coverage of CGM with decreased documentation burden, and improved access to diabetes self-management education [58]. Additionally, provider education [16] is critical to optimize prescribing, use, and overcoming provider discomfort with technology [58].

### Strengths and Limitations

This study examines the experiences of adults with T2DM using CGM in primary care, most of whom had public insurance (Medicare or Medicaid). Including participants with varied disease severity, insurance type, medication regimens, and CGM use durations provides insights into factors relevant to the complexity of primary care clinics.

Recruitment primarily occurred through primary care physician outreach, but many patients were unreachable or missed scheduled sessions, reflecting the complexity of their lives in which diabetes management is only 1 concern. Convenience sampling underrepresented patients less engaged in care or those who discontinued CGM. Thus, findings are not generalizable for less engaged patients, warranting further study. Purposive sampling was preferred but infeasible due to inaccurate contact information and resource constraints. While it was our intention to complete 3 focus groups, participants opted for the convenience of scheduling interviews.

Although participants received diabetes care in primary care, some may have initially obtained CGM from endocrinology. However, only 2 had endocrinology visits in the past year, suggesting minimal ongoing influence.

### Conclusions

Our findings highlight that while adults with T2DM highly value CGM for diabetes management, opportunities to optimize the experience, support, and use of CGM in primary care are numerous. While affirming barriers well-described in other populations in the literature, this study uniquely demonstrates the impact of comorbidities, the trust in CGM data compared to glucometer monitoring, and the reliance on receivers to use CGM technology in an adult T2DM population in primary care.

Successful use of CGM in primary care will require efforts around (1) assessing a patient’s openness and ability to access CGM, (2) developing initiation processes, (3) supporting ongoing troubleshooting, including alarm optimization, and (4) improving the use of data from CGM. The results of this study provide valuable insight for enhancing CGM use for adults with T2DM in primary care by supporting future intervention trials.

## Supplementary material

10.2196/73446 Multimedia Appendix 1 Session questions.

10.2196/73446 Multimedia Appendix 2 Code book.

10.2196/73446 Checklist 1 COREQ checklist.

## Abbreviations

CGM: continuous glucose monitor
COREQ: Consolidated Criteria for Reporting Qualitative Research
EHR: electronic health record
ESC: University of Cincinnati Evaluation Service Center
HbA_1c_: hemoglobin A_1c_
HBM: Health Belief Model
T2DM: type 2 diabetes mellitus
TAM: Technology Acceptance Model
