# Supplementary material for: Continuous Glucose Monitors Among Adults With Type 2 Diabetes Mellitus in the Primary Care Setting: Qualitative Study Informed by Technology Acceptance Model and Health Belief Model
Source: JMIR Diabetes. 2025 Dec 30;10:e73446. doi: 10.2196/73446 (PMC12753101; doi:10.2196/73446)
Supplement: Multimedia Appendix 1 [file diabetes-v10-e73446-s001.pdf]

Appendix 1 – Session Questions

Question	Technology Acceptance Model Component(s)	Health Belief Model Component(s)
**Interview**		
Can you tell me about yourself and your diabetes journey?	External Variables	Perceived Susceptibility, Perceived Severity
Can you walk me through how you take care of and manage your diabetes?	Perceived Usefulness, Actual System Use	Self-Efficacy, Perceived Benefits
Walk me through when you first found out about Continuous Glucose Monitors (CGM) to today. What has the journey been like?	External Variables, Perceived Usefulness, Attitude Toward Using, Actual System Use	Cues to Action, Perceived Benefits
Starting: Tell me a little bit about first starting to use CGM.	External Variables, Perceived Ease of Use, Attitude Toward Using	Cues to Action, Perceived Benefits, Perceived Barriers
Using: What is your usual day using your CGM like?	Perceived Usefulness, Actual System Use	Self-Efficacy
Stopping: Tell me about a time you stopped or took a break from using your CGM.	Attitude Toward Using, Behavioral Intention to Use, Actual System Use	Perceived Barriers
Talk about how members of your healthcare team affected your use of CGM, if at all?	External Variables, Behavioral Intention to Use	Cues to Action, Perceived Benefits
What would you tell a friend with diabetes who may be thinking about using CGM?	Attitude Towards Using, Behavioral Intention to Use	Perceived Benefits, Cues to Action
**Focus Group**		
Walk me through when you first found out about Continuous Glucose Monitors (CGM) to today. What has the journey been like?	External Variables, Perceived Usefulness, Attitude Toward Using, Actual System Use	Cues to Action
How has CGM influenced the way you take care of your diabetes or your daily routines?	Perceived Usefulness, Actual System Use	Perceived Benefits, Self-Efficacy
Were there times when using CGM felt tricky or hard? Tell me about that.	Perceived Ease of Use	Perceived Barriers
What could make your experience with CGM better?	Perceived Ease of Use	Perceived Barriers, Self-Efficacy
