# Supplementary material for: Continuous Glucose Monitors Among Adults With Type 2 Diabetes Mellitus in the Primary Care Setting: Qualitative Study Informed by Technology Acceptance Model and Health Belief Model
Source: JMIR Diabetes. 2025 Dec 30;10:e73446. doi: 10.2196/73446 (PMC12753101; doi:10.2196/73446)
Supplement: Multimedia Appendix 2 [file diabetes-v10-e73446-s002.pdf]

Appendix Table 2 – Code Book

Health Belief Model		
	Perceived Susceptibility	This refers to a person's subjective perception of the risk of acquiring an illness or disease.<br>Examples: family history of diabetes, knew it would happen at some point, story of diagnosis
	Perceived Severity	This refers to a person's feelings on the seriousness of contracting or failing to manage diabetes. This can include medical consequences (e.g., death, disability) and social consequences (e.g., family life, social relationships).<br>Examples: unable to control not matter the medications, stories about high or low blood glucose, "beating it"
	Perceived Barriers	This refers to a person's feelings on the obstacles to using CGM or managing diabetes. There is wide variation in a person's feelings of barriers, or impediments, which lead to a cost/benefit analysis. The person weighs the effectiveness of the actions against the perceptions that it may be expensive, dangerous (e.g., side effects), unpleasant (e.g., painful), time-consuming, or inconvenient.<br>Examples: technology barriers, education, insurance, physical ability, social support
	Perceived Benefits	Participants' views on the benefits of using CGMs for monitoring glucose levels and reducing diabetes burden and disease.<br>Examples: reminder, less stressful, removes doubt, less painful, no finger sticks
	Cues to Action	The intrinsic and external stimulus that triggers improved diabetes management.<br>Examples: people providing support, reminders, education, finding resources
Technology Acceptance Model		
	Perceived Usefulness (functional benefits)	The extent to which individuals believe that using CGM will provide functional benefits in managing their diabetes, ie helps them manage their disease and makes life easier.<br>Example: not doing finger sticks, managing lows, better guidance to take right dose of medicine, reaction to individual numbers from CGM
	Perceived ease-of-use (user-friendliness)	The extent to which individuals find CGM user-friendly and easy to use.<br>This includes examples of what make it not easy to use or difficulties the patient faced.

	External Variables	Factors external to the individual that may influence their acceptance and use of CGM. This may include social environment, external support, societal influences, or technological infrastructure.<br>Ex. insurance, ease of refills when supplies broken, doctor recommended, pharmacist taught me, family member helped apply and set-up
	Attitude Toward Using	The individual's overall positive or negative feelings and evaluations regarding use of CGM for diabetes management.<br>Examples: not that bad to start, had to play with it to figure it out, would highly recommend it, convenient, wishes it could do x
	Behavioral Intention to Use	The individual's subjective likelihood or willingness to adopt and use a technology in the future or continue to use after starting. Focuses on future and planning around disease management.
Attitude - diabetes medications		The individual's overall positive or negative feelings and evaluations regarding diabetes medications.
Self-efficacy		A person's confidence in his or her ability to successfully manage diabetes and use CGM. Includes stories about disease management.<br>Examples: previous education/training, systems for managing, tech literacy
Behavior Change		Changes in behavior, actions, or habits as a result of CGM use.<br>Examples: diet changes, medication changes
Exercise		Capture conversations around activity levels and exercise.
Diet		Capture general thoughts about eating habits including general observation about habits, changes, adjustments, and learning about diet.
Facilitators		Factors that helped make the use of CGM easier. The things that patient figured out to make this work.<br>Examples: people, changes, social factors, comfort with technology, etc
Social Commentary		General comments on diabetes, American diet and technology for disease management. Not reflective of the patients story but a reflection of the patient onto society. Beliefs and mistrust about healthcare in general.

Diabetes Monitoring	Comments and reactions to physician monitoring of diabetes and management (HbA1c, lab work, other clinical measures)<br>EXCLUDES conversation about individual diabetes numbers readings and patient self-management
Co-morbidities	Diabetes does not exist in isolation. Capture discussion around the impact of other disease states on diabetes management.<br>Examples: depression, heart disease, heart attacks
Weight	Conversation about weight and impact on overall health, severity of disease, management of trying to lose or gain weight.
