# Supplementary material for: Continuous Glucose Monitors Among Adults With Type 2 Diabetes Mellitus in the Primary Care Setting: Qualitative Study Informed by Technology Acceptance Model and Health Belief Model
Source: JMIR Diabetes. 2025 Dec 30;10:e73446. doi: 10.2196/73446 (PMC12753101; doi:10.2196/73446)
Supplement: Checklist 1 [file diabetes-v10-e73446-s003.pdf]

COREQ Checklist

No	Item	Guide questions/description	Location in Manuscript
**Domain 1: Research team and reflexivity**			
Personal Characteristics			
1	Interviewer/facilitator	Which author/s conducted the interview or focus group?	Data Collection
2	Credentials	What were the researcher's credentials? *E.g. PhD, MD*	Author Line
3	Occupation	What was their occupation at the time of the study?	Research Team
4	Gender	Was the researcher male or female?	N/A
5	Experience and training	What experience or training did the researcher have?	Research Team
Relationship with participants			
6	Relationship established	Was a relationship established prior to study commencement?	Research Team
7	Participant knowledge of the interviewer	What did the participants know about the researcher? e.*g. personal goals, reasons for doing the research*	Ethical Considerations
8	Interviewer characteristics	What characteristics were reported about the interviewer/facilitator? e.g. *Bias, assumptions, reasons and interests in the research topic*	Research Team/Ethical Considerations
**Domain 2: study design**			
Theoretical framework			
9	Methodological orientation and Theory	What methodological orientation was stated to underpin the study? *e.g. grounded theory, discourse analysis, ethnography, phenomenology, content analysis*	Study Design
Participant selection			
10	Sampling	How were participants selected? *e.g. purposive, convenience, consecutive, snowball*	Setting/ Recruitment
11	Method of approach	How were participants approached? e.*g. face-to-face, telephone, mail, email*	Rercruitment
12	Sample size	How many participants were in the study?	Results
13	Non-participation	How many people refused to participate or dropped out? Reasons?	Results
Setting			
14	Setting of data collection	Where was the data collected? e.*g. home, clinic, workplace*	Setting
15	Presence of non-participants	Was anyone else present besides the participants and researchers?	Data Collection
16	Description of sample	What are the important characteristics of the sample? *e.g. demographic data, date*	Table 1, Results
Data collection			
17	Interview guide	Were questions, prompts, guides provided by the authors? Was it pilot tested?	Appendix 1, Data Collection

18	Repeat interviews	Were repeat interviews carried out? If yes, how many?	Data Collection
19	Audio/visual recording	Did the research use audio or visual recording to collect the data?	Data Collection
20	Field notes	Were field notes made during and/or after the interview or focus group?	Data Collection
21	Duration	What was the duration of the interviews or focus group?	Data Collection
22	Data saturation	Was data saturation discussed?	Data Collection
23	Transcripts returned	Were transcripts returned to participants for comment and/or correction?	Data Collection
**Domain 3: analysis and findings**			
Data analysis			
24	Number of data coders	How many data coders coded the data?	Data Analysis
25	Description of the coding tree	Did authors provide a description of the coding tree?	Appendix 3
26	Derivation of themes	Were themes identified in advance or derived from the data?	Data Analysis
27	Software	What software, if applicable, was used to manage the data?	Data Analysis
28	Participant checking	Did participants provide feedback on the findings?	Data Analysis
Reporting			
29	Quotations presented	Were participant quotations presented to illustrate the themes / findings? Was each quotation identified? e*.g. participant number*	Results
30	Data and findings consistent	Was there consistency between the data presented and the findings?	Results/Discussion
31	Clarity of major themes	Were major themes clearly presented in the findings?	Results
32	Clarity of minor themes	Is there a description of diverse cases or discussion of minor themes?	Results
